# Mechanism of recipient cell-dependent differences in exosome uptake

**DOI:** 10.1186/s12885-017-3958-1

**Published:** 2018-01-06

**Authors:** Sayo Horibe, Toshihito Tanahashi, Shoji Kawauchi, Yoshiki Murakami, Yoshiyuki Rikitake

**Affiliations:** 1 Department of Medical Pharmaceutics, Kobe Pharmaceutical University, 4-19-1, Motoyamakita-machi, Higashinada-ku, Kobe, 658-8558 Japan; 2 Division of Gastroenterology, Department of Internal Medicine, Kobe University Graduate School of Medicine, 7-5-1 Kusunoki-cho, Chuo-ku, Kobe, 650-0017 Japan; 3 Educational Center for Clinical Pharmacy, Kobe Pharmaceutical University, 4-19-1 Motoyamakita-machi, Higashinada-ku, Kobe, 658-8558 Japan; 4 Department of Hepatology, Graduate School of Medicine, Osaka City University, 1-4-3 Asahi-machi, Abeno-ku, Osaka, 545-8585 Japan

**Keywords:** Exosome, Endocytosis, Caveolae, Clathrin, Carcinoma cell

## Abstract

**Background:**

Exosomes, small-membrane vesicles, are secreted by cells and include several types of proteins and nucleic acids. Exosomes transfer cellular information derived from donor cells and are involved in various physiological and pathological events, such as organ-specific metastasis. Elucidating the exosome uptake mechanisms is important for understanding the progression processes of organ-specific metastasis. However, whether the exosomes secreted by the donor cells are selectively or non-selectively incorporated into the recipient cells is unknown.

**Methods:**

In this study, three human carcinoma cell lines, A549 (lung), HCT116 and COLO205 (colon), were used. The exosome isolation efficiency was compared between three methods: ultracentrifugation, ExoQuick-TC and Total Exosome Isolation kits. Recipient cells were treated with Pitstop 2, an inhibitor of clathrin-dependent endocytosis, or genistein, an inhibitor of caveolae-dependent endocytosis, and then incubated with DiO-labeled exosomes.

**Results:**

Among the three methods examined, ultracentrifugation was the most efficient and reproducible. Exosomes derived from a donor cell line are incorporated into the three cell lines, but the exosome uptake capability was different depending on the recipient cell type and did not depend on the donor cell type. Exosome uptake in COLO205 was inhibited by Pitstop 2 and genistein. Exosome uptake in HCT116 was inhibited by Pitstop 2, but not genistein, while that in A549 cells was not inhibited by these inhibitors. Taken together, these results suggest that the exosomes secreted by donor cells are non-selectively incorporated into recipient cells and that the exosome uptake mechanism is different depending on the recipient cells.

**Conclusions:**

Different recipient cells’ exosome uptake capabilities may be involved in organ-specific metastasis.

## Background

Exosomes are small-membrane vesicles (30–100 nm in diameter) secreted by cells. They contain different types of functional molecules, including proteins such as tetraspanins and nucleic acids such as DNAs, mRNAs and microRNAs (miRNAs), depending on the cell type [1]. Exosomes are found in almost all physiological fluids including urine, plasma, saliva, serum and breast milk, and circulate throughout the whole body. The molecules included in exosomes are potential diagnostic biomarkers of disease [2]. Exosomes secreted by cells are incorporated into recipient cells, which receive information from the donor cells and exchange functions. Therefore, exosomes have emerged as important mediators of cell–cell communication involved in various physiological and pathological conditions, such as progression of cancer [3], liver disease [4], immune-defective disease [5] and neurodegenerative disease [6]. It has been reported that uptake of exosomes released by colon cancer cells induces tumor-like transformation in human colon-derived mesenchymal stem cells [7] and that exosomes derived from cancer cells and microglia are relevant to cancer metastasis [8]. Peinado and colleagues reported that melanoma cell-derived exosomes could be transferred only to the lung [9]. Thus, the cell–cell communication mediated by exosomes may be involved in organ-specific metastasis. However, whether the exosomes secreted by donor cells are selectively or non-selectively incorporated into recipient cells is unknown.

Recently, it has been reported that the isoforms of integrin expressed in exosomes contribute to the delivery of the exosomes to specific organs and tissues, which have the uptake property of exosomes derived from various cancer cells. However, the mechanism by which exosomes are incorporated into specific organs and tissues is unknown. Therefore, it is important to elucidate how exosomes are incorporated into individual cells after delivery to specific organs and tissues [10].

It is conceivable that endocytosis is the main mechanism of exosome uptake [11]. Endocytosis can be divided into at least four pathways, including caveolae-dependent endocytosis, clathrin-dependent endocytosis, macropinocytosis and phagocytosis [12], and these pathways have been reported to be relevant to exosome uptake [13]. It can be expected that elucidating the exosome uptake mechanism in individual organs and tissues will contribute to restraining the exosome-mediated progression and/or metastasis of cancer. However, whether the exosome uptake mechanism is different depending on tissues is unknown, and the underlying mechanism of the tissue-dependent exosome uptake remains unidentified. Here, we attempted to elucidate the exosome uptake mechanism in human lung carcinoma cell line A549 and human colon carcinoma cell lines HCT116 and COLO205.

## Methods

### Cells and culture

Human lung carcinoma cell line A549 (RCB0098) and human colon carcinoma cell lines HCT116 (RCB2979) and COLO205 (RCB2127) were provided by the RIKEN BRC through the National Bio-Resource Project of the MEXT, Japan. A549 and HCT116 cells were cultured in Dulbecco’s modified Eagle’s medium (DMEM). COLO205 cells were cultured in RPMI 1640 medium. The cells were maintained in their respective culture media supplemented with 10% fetal bovine serum (FBS), 100 units/ml penicillin and 100 μg/ml streptomycin at 37 °C in 5% CO_2_ and 95% air.

### Exosome isolation

Exosomes were prepared by the standard ultracentrifugation method according to a previous report [14], and this method was performed as we reported previously [15]. In brief, A549 and HCT116 cells (3 × 10^6^ cells) were seeded in a 100-mm dish in culture medium with 10% FBS. After 24 h, culture medium was changed to culture medium without FBS and incubated. COLO205 cells (6 × 10^6^ cells) were seeded in a 100-mm dish in culture medium without 10% FBS and incubated. The culture media were collected after 72 h incubation and the exosomes were isolated by the following three methods: ultracentrifugation, ExoQuick-TC® (System Biosciences Inc., Palo Alto, CA, USA) and Total Exosome Isolation® (Thermo Fisher Scientific Inc., Waltham, MA, USA). The ultracentrifugation method was performed as follows. The collected medium was centrifuged at 2000 *g* for 30 min, and then at 10,000 *g* for 30 min to remove cell debris. The supernatant was centrifuged at 100,000 *g* for 70 min to purify exosomes. The pellet was washed with PBS and ultracentrifuged at 100,000 *g* for 70 min again. The pellet was resuspended with PBS and stored until use. Exosome isolation using ExoQuick-TC and Total Exosome Isolation was performed according to the manufacturer’s instructions. In brief, the collected medium was centrifuged at 2000 *g* for 30 min and supernatant was collected. One-fifth of ExoQuick-TC Exosome Precipitation Solution or half of Total Exosome Isolation were added to the supernatant and their suspension was incubated overnight at 4 °C. The suspension was centrifuged at 1500 *g* for 30 min for ExoQuick-TC or at 10,000 *g* for 60 min for Total Exosome Isolation. The pellet was resuspended with PBS. Exosome protein content was qualified using the BCA protein assay kit (Thermo Fisher Scientific) before further experiments.

### Uptake of DiO-labeled exosomes by recipient cells

Twenty-four μg of exosomes were incubated with lipophilic tracer DiO solution (Thermo Fisher Scientific) for 20 min at 37 °C. Excessive DiO was removed with Exosome Spin Columns (MW 3000) (Thermo Fisher Scientific). Exosome labeling efficiency was analyzed with an Infinite® 200 PRO fluorometer (TECAN, Männedorf, CHE). The cells were seeded in an 8-well chamber slide (1 × 10^4^ or 4 × 10^4^ cells/well) and incubated for 24 h. DiO-labeled exosomes (8 μg) were added to the culture media of the recipient cells and incubated for 3 h at 37 °C. The recipient cells were fixed with 4% paraformaldehyde at room temperature for 10 min and permeabilized with 0.1% Triton X-100 at room temperature for 5 min. The cells were stained with Alexa Fluor 555 phalloidin (Thermo Fisher Scientific) at room temperature for 30 min and mounted in Prolong® Diamond Antifade Reagent with DAPI (Thermo Fisher Scientific), and the slide was covered with cover glass. The cells were visualized with an EVOS FL fluorescence microscope (Thermo Fisher Scientific).

### Total RNA extraction from cell lines

Total RNA was extracted from cell pellets using TRIzol reagent (Thermo Fisher Scientific), according to the manufacturer’s instructions. In brief, the cells were lysed by TRIzol and chloroform was added to the cell lysis. The suspension was centrifuged at 12,000 *g* for 15 min and aqueous phase was collected. Isopropyl alcohol was added to the aqueous phase and then was centrifuged at 12,000 *g* for 10 min. The supernatant was removed and 75% ethanol was added to the pellet for washing RNA. The suspension was centrifuged at 7500 *g* for 10 min and the supernatant was removed. The pellet was dissolved by RNase-free water. The quantity of total RNA was determined using an ND-1000 spectrophotometer (NanoDrop Technologies, Wilmington, DE, USA).

### Quantitative real-time PCR

Total RNA (0.2 μg) from each sample was reverse transcribed to complementary DNA (cDNA) for real-time PCR using a ReverTra Ace qPCR RT Kit (Toyobo, Osaka, Japan), according to the manufacturer’s protocol. In brief, the reaction was conducted by incubating for 10 min at 25 °C followed by 60 min at 42 °C and 5 min at 95 °C. PCR reaction was monitored in real-time with a Thermal Cycler Dice Real Time System (TaKaRa Bio, Otsu, Japan). The PCR reaction was carried out in 20 μl of a reaction mixture composed of Thunderbird SYBR qPCR Mix (Toyobo) and 0.5 μM of each primer. The reaction mixture was subjected to an initial denaturation at 95 °C for 20 s, followed by 50 cycles of amplification at 95 °C (3 s) for denaturation, and at 60 °C (30 s) for annealing. After the cycles, a melting curve was checked to confirm the single product. Relative expression levels of target genes were calculated by the delta-delta Ct method with Glyceraldehyde-3-phosphate dehydrogenase (GAPDH) used as a reference gene. The PCR primer sequences used for detecting gene expression of clathrin, caveolin-1 and GAPDH were as follows: clathrin forward 5’-GTTACTGCACCTCATGAAGCC-3′ and reverse 5’-AGTTCTTCAGCACCGGCTAA-3′; caveolin-1 forward 5’-GTCAACCGCGACCCTAAACA-3′ and reverse 5’-GATGCCAAAGAGGGCAGACA-3′; GAPDH forward 5’-TTCTTTTGCGTCGCCAGCCGA-3′ and reverse 5’-GTGACCAGGCGCCCAATACGA-3′.

### Whole-cell protein extracts

Cells were lysed with RIPA buffer (Wako, Osaka, Japan), composed of 50 mM Tris-HCl (pH 7.4), 1% NP-40, 0.5% SDC, 0.1% SDS, 150 mM NaCl, and protease inhibitor cocktail (Nacalai tesque, Kyoto, Japan). The supernatants obtained after centrifugation at 15,000 *g* for 10 min at 4 °C were used as whole-cell protein extracts.

### Western blotting

Total exosome protein (2 μg) was resuspended by 5× RIPA buffer (125 mM Tris-HCl, 750 mM NaCl, and 5% NP-40, 5% sodium deoxycholate and 0.5% SDS) and then suspension was sonicated for 5 min and incubated for 15 min on ice. The suspension of whole-cell protein extracts (20 μg) was boiled in a sixth-volume of sample buffer (Nacalai tesque) and separated on 12% SDS-polyacrylamide gels. Proteins on the gels were transferred to polyvinylidene difluoride membranes, which were blocked with Blocking One (Nacalai tesque) overnight at 4 °C. Anti-CD63 rabbit polyclonal antibody (1:200; System Biosciences Inc.), anti-CD9 rabbit polyclonal antibody (1:200; System Biosciences Inc.), anti-CD81 rabbit polyclonal antibody (1:200; System Biosciences Inc.), anti-HSP70 rabbit polyclonal antibody (1:500; System Biosciences Inc.), anti-clathrin mouse polyclonal antibody (1:2000; BD Biosciences, Franklin Lakes, NJ, USA), anti-caveolin-1 mouse monoclonal antibody (1:500; BD Biosciences) and anti-β-actin rabbit monoclonal antibody (1:5,000; Cell Signaling Technology, Danvers, MA, USA) were used as primary antibodies. The membranes were subsequently incubated with horseradish peroxidase-conjugated secondary antibodies for 1 h at room temperature. The secondary anti-rabbit IgG or the secondary anti-mouse IgG was diluted to 1:5,000–1:10,000 or 1:5,000, respectively. Protein/antibody complexes were visualized with Chemi-Lumi One Super (Nacalai tesque) and detected using an Image Quant LAS 4000 (GE Healthcare Biosciences, Piscataway, NJ, USA).

## Results

### Most efficient enrichment of exosome marker proteins by the ultracentrifugation method

**Fig. 1:**
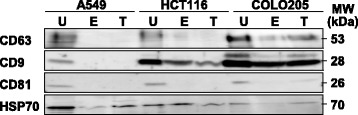
Most efficient enrichment of exosome marker proteins by ultracentrifugation method. Exosomes were isolated from A549, HCT116 and COLO205 cells using the ultracentrifugation, ExoQuick-TC™ and Total Exosome Isolation methods. Expression of CD63, CD9, CD81 and HSP70 was examined by western blotting using specific antibodies. Representative results are shown. U, ultracentrifugation; E, ExoQuick-TC™; T, Total Exosome Isolation kits

CD63, CD9, CD81 and HSP70 are well-known marker proteins of exosome membrane [16, 17]. Using the ExoQuick-TC and Total Exosome Isolation kits, CD63 expression was not or hardly detected in A549- and HCT116-derived exosomes but was detected in COLO205-derived exosomes (Fig. 1). Similarly, CD9 expression was not detected in A549-derived exosomes but was detected in HCT116- and COLO205-derived exosomes. CD81 expression was not detected, whereas HSP70 expression was detected in exosomes derived from all the cell lines. Using the ultracentrifugation method, expression of all four marker proteins was detected in exosomes derived from all the cell lines, and their expression levels were greater than those isolated using the ExoQuick-TC and Total Exosome Isolation kits. Thus, the ultracentrifugation method was the most efficient and reproducible.

CD63 expression in COLO205-derived exosomes was higher than that in A549- and HCT116-derived exosomes. CD9 expression was increased in the order of COLO205, HCT116 and A549 cells. CD81 expression was equivalent among these three cell lines. HSP70 expression was highest in A549-derived exosomes. These results indicate that the expression profile of the exosome marker proteins was different depending on the donor cells.

### Uptake of DiO-labeled A549-, HCT116- and COLO205-derived exosomes

**Fig. 2:**
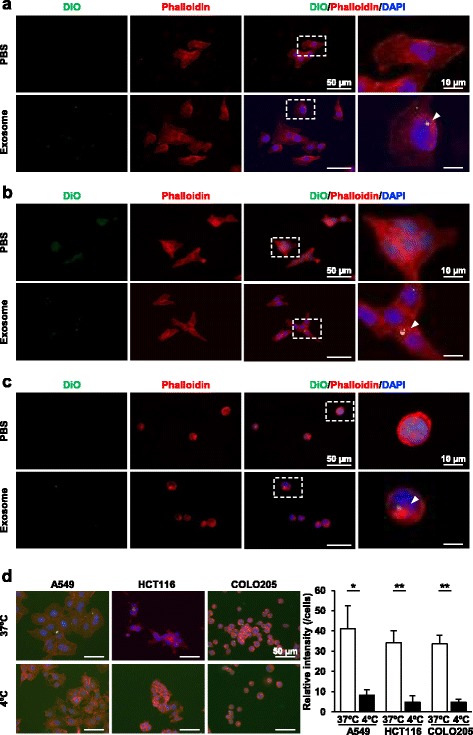
Uptake of DiO-labeled A549-, HCT116- and COLO205-derived exosomes into the individual donor cells. Exosomes were isolated from A549 (**a**), HCT116 (**b**) and COLO205 (**c**) cells using the ultracentrifugation method. Uptake of DiO-labeled exosomes at 37 °C (**a-d**) or 4 °C (**d**) was analyzed by immunofluorescence microscopy. Representative images are shown. Magnified images represent high-magnification images of the boxed area (**a-c**). Immunofluorescence intensities of DiO-labeled exosomes per cell were analyzed using ImageJ software (**d**). Data are expressed as means ± SEM (*n* = 5). *P < 0.05; **P < 0.01; NS: not significant

To maintain cell homeostasis, exosomes derived from donor cells must be incorporated into the donor cells themselves in a paracrine mechanism [18]. We therefore investigated whether exosomes derived from the donor cells were efficiently incorporated into the recipient cells, when the donor and recipient cells were the same cell type. DiO-labeled A549-derived exosomes were incorporated into A549 cells (Fig. 2a). The same is true of DiO-labeled HCT116- and COLO205-derived exosomes (Fig. 2b and c). The exosome uptake levels at 4 °C were much lower than those at 37 °C, and the exosome uptake levels at 4 °C in A549, HCT116 and COLO205 cells were similar (Fig. 2d). These results indicate that exosomes derived from the donor cells are incorporated into the donor cells themselves and that this uptake is energy-dependent.

**Fig. 3:**
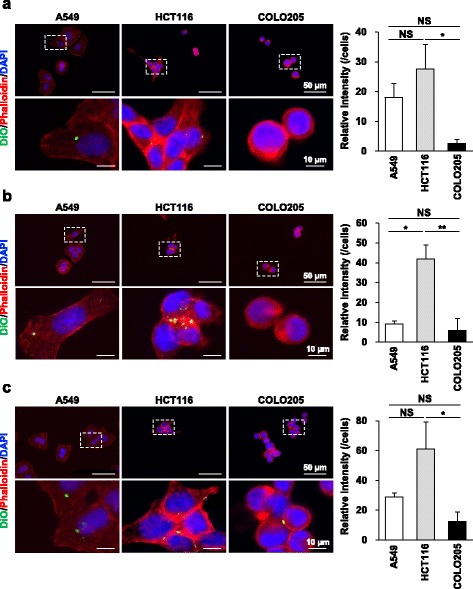
Uptake of DiO-labeled A549-, HCT116- and COLO205-derived exosomes into the individual recipient cells. Exosomes were isolated from A549 (**a**), HCT116 (**b**) and COLO205 (**c**) cells using the ultracentrifugation method. Uptake of DiO-labeled exosomes was analyzed by immunofluorescence microscopy. Representative images are shown. Magnified images represent high-magnification images of the boxed area. Immunofluorescence intensities of DiO-labeled exosomes per cell were analyzed using ImageJ software. Data are expressed as means ± SEM (*n* = 3–5). *P < 0.05; **P < 0.01; NS: not significant

We then compared the exosome uptake capability in A549, HCT116 and COLO205 cells and found that DiO-labeled exosome uptake was increased in the order of HCT116, A549 and COLO205 cells, irrespective of the donor cell type (Fig. 3a-c). These results indicate that exosome uptake capability is different depending on the recipient cell type and does not depend on the donor cell type.

### Expression of endocytosis-related caveolin-1 and clathrin in recipient cells

**Fig. 4:**
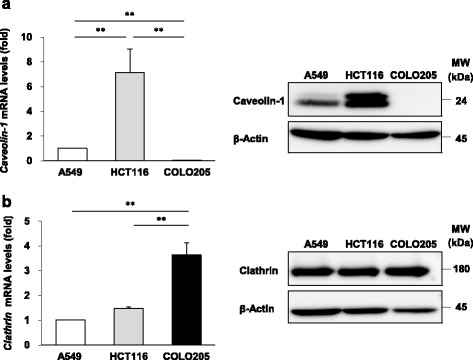
Expression of endocytosis-related caveolin-1 and clathrin in recipient cells. mRNA expression of caveolin-1 (**a**) and clathrin (**b**) was examined by real-time PCR and protein expression of caveolin-1 (**a**) and clathrin (**b**) was examined by western blotting using specific antibodies. Representative results of western blotting are shown. Data are expressed as means ± SEM (*n* = 3). **P < 0.01

Caveolin-1 and clathrin heavy chain are critical to mediate caveolae-dependent endocytosis and clathrin-dependent endocytosis, respectively. To elucidate the cause of the different exosome uptake capabilities, we examined the expression of caveolin-1 and clathrin in recipient cells. Caveolin-1 mRNA and protein levels in HCT116 cells were higher than those in A549 cells (Fig. 4a). Caveolin-1 mRNA and protein were not detected in COLO205 cells (Fig. 4a). Meanwhile, clathrin mRNA levels in COLO205 cells were higher than those in A549 and HCT116 cells, although clathrin protein levels were equivalent among these three cell lines (Fig. 4b). These results may imply that efficient exosome uptake is associated with abundant caveolin-1 expression in HCT116 cells.

### Different exosome uptake mechanisms among the three cell lines

**Fig. 5:**
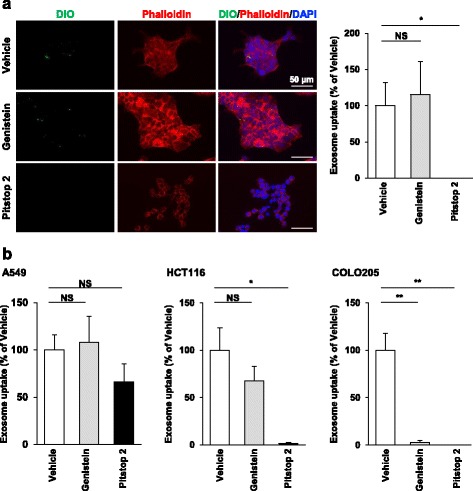
Different exosome uptake mechanisms among the three cell lines. Exosomes were isolated from COLO205 (**a**) and A549 cells (**b**) using the ultracentrifugation method. A549, HCT116 and COLO205 cells were treated with 200 μM genistein or 10 μM Pitstop 2 for 10 min and then incubated with DiO-labeled exosomes for 3 h. Uptake of DiO-labeled exosomes was analyzed by immunofluorescence microscopy. Representative images are shown. Immunofluorescence intensities of DiO-labeled exosomes per cell were analyzed using ImageJ software. Data are shown as the percentage of exosome uptake relative to the vehicle-treated cells and are expressed as means ± SEM (*n* = 5–10). *P < 0.05; **P < 0.01; NS: not significant

To evaluate whether efficient exosome uptake is associated with abundant caveolin-1 expression in HCT116 cells, we examined the effect of genistein, an inhibitor of caveolae-dependent endocytosis [19]. Treatment of HCT116 cells with genistein had little or no effect on uptake of DiO-labeled COLO205-derived exosomes (Fig. 5a). We then examined the effect of Pitstop 2, an inhibitor of clathrin-dependent endocytosis [20]. Treatment of HCT116 cells with Pitstop 2 inhibited uptake of DiO-labeled COLO205-derived exosomes (Fig. 5a). These results indicate that efficient exosome uptake in HCT116 cells is mediated by clathrin-dependent endocytosis but not by caveolae-dependent endocytosis.

To clarify whether the exosome uptake mechanism in A549 and COLO205 cells is the same as that in HCT116 cells, we examined the effects of Pitstop 2 and genistein on uptake of DiO-labeled A549-derived exosomes in these three cell lines. Treatment of HCT116 cells with Pitstop 2, but not with genistein, inhibited uptake of DiO-labeled A549-derived exosomes (Fig. 5b). Treatment of COLO205 cells with these two inhibitors inhibited uptake of DiO-labeled A549-derived exosomes, whereas they did not inhibit it in A549 cells (Fig. 5b). These results indicate that both clathrin-dependent and caveolae-dependent endocytosis are involved in COLO205 cells, and clathrin-dependent but not caveolae-dependent endocytosis is involved in HCT116 cells, but neither of them is involved in A549. Thus, the exosome uptake mechanism is different depending on the recipient cells.

## Discussion

Exosome isolation methods include ultracentrifugation and density-gradient centrifugation, but their techniques are relatively complicated [14]. Currently, the most widely used method for exosome isolation is ultracentrifugation, which, in its classical form, consists of multiple centrifugation steps with increasing centrifugal strength to sequentially pellet cells (300 *g*), cell debris (10,000 *g*) and exosomes (100,000 *g*). In addition to these traditional isolation methods, easy-to-use precipitation solutions such as ExoQuick and Total Exosome Isolation have been commercialized in the last few years with no need for expensive equipment or skillful techniques. Moreover, methods using these kits have an advantage in that they can purify exosomes from lower volumes of cell culture media and blood than the ultracentrifugation method. Although their mode of action has not been disclosed, these kits are commonly used. However, we observed no or less expression of exosome marker proteins such as CD63, CD9, CD81 and HSP70 in isolated exosomes using the kits than those using the ultracentrifugation method. Exosome isolation methods using the kits binding to a water molecule reduce water solubility of microvesicles in the samples. Therefore, the microvesicles except exosomes are co-isolated, leading to apparent increases in collected protein mass in isolated exosome using the kits. Accordingly, exosome marker protein expression using these kits was lower than that using the ultracentrifugation method. In conducting research on exosomes, it is important that exosomes can be easily and reproducibly isolated. It is necessary to establish a method capable of constantly isolating exosomes to identify the exosome uptake mechanism. Therefore, we employed the ultracentrifugation method.

We showed here that exosome marker protein expression was different among the three carcinoma cell lines. Consistent with our results, exosome marker protein expression was different among B-cell lymphoma cell lines [21]. Proteins present on the surface of exosomes have been reported to influence the exosome’s uptake rate into the recipient cells [22]. Therefore, one may assume that different exosomal membrane protein expression could play a role in tissue-selective exosome uptake. We showed here that exosomes were incorporated into both donor and recipient cells and that irrespective of the donor cells, the exosome uptake amount was largest in HCT116 cells. Exosome uptake is facilitated by the attachment of a cell-penetrating peptide to exosomes [23]. However, the exosome uptake levels at 4 °C were similar between A549, HCT116 and COLO205 cells. Accordingly, the apparent uptake observed at 4 °C may reflect the surface binding of DiO-labeled exosomes. Therefore, highly capable exosome uptake in HCT116 is not dependent on membrane permeability. These results suggest that exosome uptake is dependent on recipient cells, but not on the surface molecules of exosomes.

It is known that exosomes are incorporated into cells by endocytosis and their uptake capability is different depending on the endocytosis-related molecule expression level. Endocytosis has caveolae-dependent and clathrin-dependent pathways, and caveolin-1 expression is involved in caveolae-dependent endocytosis. We showed here that caveolin-1 expression was greater in the order of COLO205, A549 and HCT116 cells, similar to the exosome uptake capability observed in this study. These results suggest that the exosome uptake capability might be correlated with the expression level of caveolin-1. However, the exosome uptake in HCT116 and A549 cells was not affected by genistein, an inhibitor of caveolae-dependent endocytosis. Meanwhile, Pitstop 2, a potent inhibitor of clathrin-dependent endocytosis, could inhibit exosome uptake in HCT116, indicating that exosome uptake is mediated by clathrin-dependent endocytosis. Moreover, we analyzed the exosome uptake mechanism and found that exosome uptake in HCT116 and COLO205 cells was mediated by clathrin-dependent endocytosis, and that in A549 cells was mediated by neither clathrin- nor caveolin-dependent endocytosis. These results indicate that the exosome uptake mechanism differs depending on the recipient cell type. Consistent with our results, clathrin-mediated endocytosis plays a role in PC12 cell-derived exosome uptake [13]. This report showed that macropinocytosis is involved in exosome uptake in other cells [13], providing further evidence that the exosome uptake mechanism is different dependent on the recipient cell type [24].

We found that exosomes derived from A549 and COLO205 cells were also incorporated into human umbilical vein endothelial cells (data not shown). This result was consistent with a previous report showing that glioblastoma-derived exosomes were incorporated into human umbilical vein endothelial cells [25]. In addition, it was recently reported that tumor extracellular acidity is important for both increasing exosome release by tumor cells and favoring exosome uptake [26, 27]. To clarify the cancer cell metastasis mechanism in the human body, it is necessary to examine whether and how exosomes derived from cancer cells are incorporated into non-cancerous cells, such as endothelial cells and fibroblasts, under in vivo conditions.

## Conclusions

Exosome uptake capability was not dependent on the expression of exosome marker proteins but on the recipient cells. Our results suggest that organ-specific metastasis mediated by exosomes is related to the different recipient cells’ exosome uptake capabilities.

## Abbreviations

DMEM: Dulbecco’s modified Eagle’s medium
GAPDH: Glyceraldehyde-3-phosphate dehydrogenase
